# Association Between Mediterranean Diet and Other Healthy Habits and Sociodemographic Variables with the Values of Vascular and Heart Age in Spanish Workers

**DOI:** 10.3390/nu17050903

**Published:** 2025-03-05

**Authors:** Natalia Montero Muñoz, Pedro Juan Tárraga López, Ángel Arturo López-González, Hernán Paublini, Cristina Martorell Sánchez, Emilio Marínez-Almoyna Rifá, José Ignacio Ramírez-Manent

**Affiliations:** 1 Family Medicine, Balearic Islands Health Service, 07122 Palma de Mallorca, Spain; nataliamonteromunoz@gmail.com (N.M.M.); joseignacio.ramirez@ibsalut.es (J.I.R.-M.); 2 Faculty of Medicine of Castilla la Mancha, 02071 Albacete, Spain; pjtarraga@sescam.jccm.es; 3 ADEMA-Health Group of IUNICS, University of Balearic Islands, 07120 Palma de Mallorca, Spain; h.paublini@eua.edu.es (H.P.); c.martorell@eua.edu.es (C.M.S.); emilio@mompra.com (E.M.-A.R.); 4 IDISBA, Balearic Islands Health Research Institute Foundation, Balearic Islands, 07004 Palma de Mallorca, Spain; 5 Faculty of Medicine of Balearic Islands, Balearic Islands University, 07122 Palma de Mallorca, Spain

**Keywords:** vascular age, heart age, Mediterranean diet, sociodemographic variables, physical activity, alcohol intake

## Abstract

**Introduction**: The assessment of cardiovascular risk has traditionally relied on validated scales designed to estimate the likelihood of experiencing a cardiovascular event within a specific timeframe. In recent years, novel methodologies have emerged, offering a more objective evaluation of this risk through indicators such as vascular age (VA) and heart age (HA). **Objective**: This study aimed to investigate the relationship between sociodemographic factors, lifestyle behaviors, and their impact on VA and HA. **Materials and Methods**: A dual study design, encompassing both cross-sectional and longitudinal retrospective approaches, was conducted among a cohort of employees. The variables assessed included sociodemographic characteristics (age, sex, and socioeconomic status) and health-related habits (smoking, physical activity, adherence to the Mediterranean diet, and alcohol consumption). **Results**: The findings revealed that all analyzed variables were significantly associated with elevated VA and HA values. Among these, age demonstrated the strongest association, with odds ratios (OR) of 114.91 (95% CI: 100.45–131.43) for high HA and 34.48 (95% CI: 31.41–37.56) for high VA. **Conclusions**: The profile of individuals most at risk for elevated VA and HA encompasses males of advanced age, characterized by low socioeconomic status, a sedentary lifestyle, poor adherence to the Mediterranean diet, and regular alcohol consumption.

## 1. Introduction

Vascular age (VA) [1,2] and heart age (HA) [3] are concepts used to assess an individual’s cardiovascular health beyond their chronological age (CA) [4]. While CA simply reflects the time a person has lived, VA and HA are more comprehensive measures that account for risk factors [5] and the health of blood vessels and the heart, respectively. These metrics are vital for predicting cardiovascular diseases and developing personalized prevention strategies.

VA refers to the condition of blood vessels, including arteries and veins, and how this condition compares to that of an average individual of the same CA. Atherosclerosis [6], arterial stiffness, and the presence of plaques in the intima [7] significantly influence VA. Individuals with a VA higher than their CA face increased risks of cardiovascular diseases, strokes, and other vascular-related complications.

HA, on the other hand, evaluates the heart’s health based on cardiovascular risk factors [8] such as blood pressure, cholesterol levels, smoking, diabetes, and obesity. This metric provides a clear perspective on the likelihood of developing heart diseases in the future.

Several tools and calculators have been developed to estimate VA and HA [9]. These tools typically use data such as blood pressure, cholesterol levels, body mass index (BMI), smoking history, and diabetes status. Determining VA and HA enables individuals and healthcare professionals to identify risks and take preventive measures, which may include lifestyle changes such as improving diet, increasing physical activity, quitting smoking, and better management of diabetes and hypertension. In some cases, medications to lower cholesterol or blood pressure may also be required.

Measuring vascular age [10] and heart age offers a more comprehensive view of cardiovascular health. Tools such as pulse wave velocity [11], ankle-brachial index [12], arterial imaging [13], cardiovascular risk calculators [14], electrocardiograms [15,16], magnetic resonance imaging (MRI) [17], and blood biomarkers [18] are essential for these assessments. By combining these methodologies, healthcare professionals can better identify risks and develop personalized prevention strategies to improve cardiovascular health and reduce the incidence of cardiovascular diseases [19].

Based on the aforementioned concepts, the objective of this study is to determine how various healthy habits (Mediterranean diet, physical activity, smoking, and alcohol consumption) and sociodemographic variables (age, gender, social class, and educational level) affect VA and HA values as determined by risk scales.

## 2. Materials and Methods

### 2.1. Participants

A descriptive cross-sectional study was conducted involving 139,634 Spanish workers (83,282 men and 56,352 women) from different autonomous regions and productive sectors during the period from January 2018 to December 2019. Additionally, a longitudinal retrospective study (2009–2019) was conducted on 40,431 workers (24,229 men and 16,202 women) selected from the initial descriptive study.

The inclusion criteria for the descriptive study were as follows:

- Aged between 18 and 69 years (working age);
- Currently employed in one of the participating companies and not on medical leave;
- Willing to participate in the study and provide data for epidemiological purposes;
- Availability of all necessary variables to calculate the different risk scales.

For the longitudinal retrospective study, workers from the descriptive study who had data available between 2009 and 2019 and who had not changed their healthy habits or sociodemographic variables were selected.

**Figure 1:**
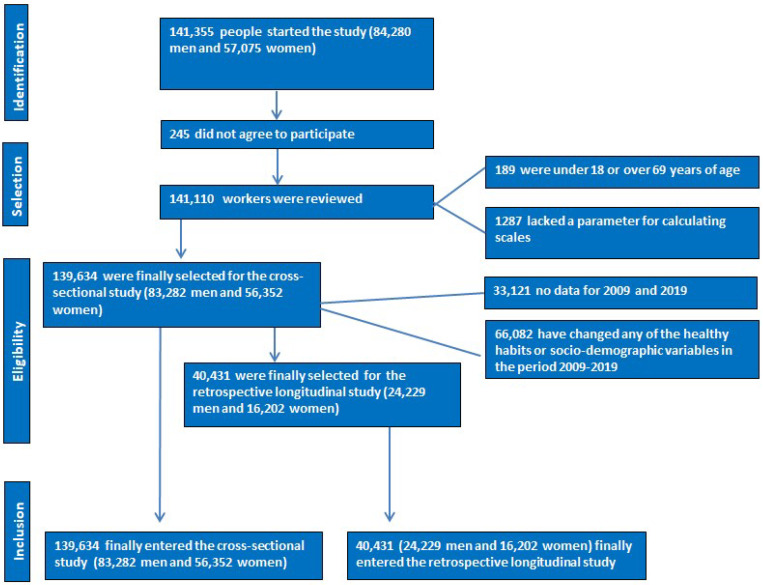
Flowchart of participants.

The flowchart for both studies is presented in Figure 1.

### 2.2. Determinations of Variables

#### 2.2.1. Anthropometric Determinations

Personnel from the several occupational health units involved in the study determined various anthropometric, clinical, and analytical factors. A SECA 700 model scale (SECA, Chino, CA, USA) with a capacity of 200 kg and 50 g divisions was used to measure weight in kilograms and height in centimeters. A SECA 220 telescopic measuring rod (SECA, Chino, CA, USA) with millimetric division and a range of 60 to 200 cm was also utilized. With the subject standing straight, feet together, trunk erect, abdomen relaxed, and upper limbs hanging on both sides of the body, the subject’s waist circumference was measured using a tape measure. At the level of the last floating rib, the tape measure was positioned parallel to the ground.

#### 2.2.2. Clinical Determinations

The subject’s blood pressure was taken while they were seated. Following a ten-minute rest period, three measurements were taken at one-minute intervals, and the average of the three measurements was found.

#### 2.2.3. Analytical Determinations

In reference laboratories, the analytical determinations were carried out following a minimum 12 h fast. Triglycerides, total cholesterol, and blood glucose were measured automatically using enzymatic techniques. By precipitating dextran sulfate Cl2Mg, HDL was ascertained. The Friedewald algorithm was used to compute LDL, assuming that triglycerides were fewer than 400 mg/dL. When triglycerides were higher than 400 mg/dL, LDL-c was obtained by direct estimation. We use mg/dL to express all values. The Friedewald formula [20] LDL is equal to total cholesterol−HDL + triglycerides/5.

#### 2.2.4. Risk Scales

If an individual had smoked one cigarette a day for the previous thirty days or had given up smoking less than a year prior, they were classified as smokers.

The social class was divided into three categories based on the 2011 national classification of occupations (CNAE) and applying the criteria of the Spanish Society of Epidemiology: class I (directors/managers, university professionals, athletes, and artists); class II (intermediate occupations and self-employed workers without salaried workers); and class III (unskilled workers). This was in accordance with the proposal of the social determinants group of the Spanish Society of Epidemiology [21].

Three categories for educational attainment were established: elementary, high school, and university.

Using the Mediterranean diet adherence Questionnaire [22], which has 14 questions with a score of 0 to 10, strong adherence is defined as results equal to or higher than 9.

The International Physical Activity Questionnaire, or IPAQ [23], was used to measure the amount of physical activity. Alcohol units (AU) are used to measure alcohol intake. One AU is equal to ten grams of pure ethanol in Spain. The weekly consumption of 14 AU for women and 21 AU for males is deemed high [24].

By applying tables utilizing several variables, such as sex, age, HDL, total cholesterol, systolic and diastolic blood pressure, tobacco use, diabetes, and antihypertensive medication, vascular age was determined using the Framingham-REGICOR model, recommended in Spain for the primary prevention of coronary heart disease [25].

The definition of avoidable lost life years (ALLY) is the difference between vascular age (VA) and chronological age (CA): VA minus CA is ALLY. Heart aging is also be covered by this idea. The cutoff of vascular age high is 18 years [26].

One measure derived from the Framingham-REGICOR cardiovascular risk scale is cardiac age.

These traditional risk scales evaluate the likelihood of experiencing a cardiac event within the following ten years, whether or not it is fatal. The following factors are taken into consideration when calculating cardiac age: age, sex, height (in cm), weight (in kg), waist circumference (in cm), diabetes, smoking (if the patient does not currently smoke, we ask if they have quit in the past year), total and HDL cholesterol, systolic blood pressure, and whether the patient is currently receiving antihypertensive medication.

HA values were found using the “Heart Age Calculator” feature on the website http://www.heartage.me, accessed on 30 December 2019. We have verified that the website is no longer available. The formula used to calculate cardiac age included total cholesterol, HDL cholesterol, fasting glucose, smoking, BMI, and systolic and diastolic blood pressure, with scores similar to the original formula. Based on these values, a series of points were added or subtracted from the chronological age, which were also adjusted by sex. The final result corresponded to the individual’s heart age. In Table 1, we provide the values assigned to each of the variables used to calculate HA. The scale can be used with people who are between 18 and 80 years old. The highest number of years that can be gained or maintained compared to chronological age (CA) is 20, while the maximum age cut-off for HA is 19 years [27].

The calculation of cardiac age using the REGICOR tool (https://regicor.cat/en/, accessed on 30 December 2019) is a clinically relevant approach to estimating an individual’s cardiovascular risk based on traditional risk factors such as blood pressure, cholesterol levels, smoking, and diabetes. It facilitates more effective communication with patients by translating cardiovascular risk into an intuitive and easily understandable concept. However, it has limitations, as it does not account for emerging factors such as chronic inflammation, insulin resistance, or body fat distribution. Additionally, its accuracy may be influenced by regional and occupational variations that affect cardiovascular risk.

### 2.3. Statistical Analysis

A descriptive analysis of categorical variables was performed by examining their frequencies and distributions. The normality of quantitative variables was evaluated using the Kolmogorov–Smirnov test, and subsequently, means and standard deviations were calculated. For the bivariate analysis, comparisons of means were conducted using Student’s *t*-test, while the chi-square test was applied to analyze proportions. Multinomial logistic regression was employed to investigate variables linked to elevated vascular and heart age values, with the model’s goodness-of-fit assessed via the Hosmer–Lemeshow test. A stratified analysis was conducted to explore potential confounding factors, but no notable confounding effects were identified. All statistical analyses were performed using SPSS software (version 29.0), with statistical significance defined as *p* < 0.05.

### 2.4. Ethical Considerations

The research team formally committed to adhering to the ethical principles outlined in the Declaration of Helsinki, with a particular emphasis on maintaining participant anonymity and ensuring the confidentiality of collected data. This study received approval from the Ethics and Research Committee of the Balearic Islands (CEI-IB) on 26 November 2020, under protocol number IB 4383/20. Participation in the study was entirely voluntary; individuals were thoroughly informed about the study’s objectives before providing both written and verbal consent. To this end, participants were given an information sheet explaining the study’s purpose, the anonymization process, and the assurance that their identities would not be disclosed in any resulting publications. The research team pledged not to share any data that could lead to the identification of participants. Furthermore, all individuals involved in the study were granted the right to access, rectify, delete, or contest the use of their personal data. The research team remains fully committed to compliance with Spain’s Organic Law 3/2018, enacted on 5 December, which governs personal data protection and the safeguarding of digital rights.

## 3. Results

The average age of the participants was slightly above 40 years. All variables, both analytical and clinical, were less favorable in men. Most individuals were between 30 and 49 years old. The vast majority belonged to social class III and had elementary education. Nearly one in three individuals was a smoker. Slightly less than 50% of women and fewer than 40% of men engaged in regular physical activity, and even fewer exhibited high adherence to the Mediterranean diet. Almost one-third of men and 15.6% of women regularly consumed alcohol. Complete data are presented in Table 2.

Table 3 presents the mean values for both vascular age (VA) and heart age (HA). It is observed that these values increase with chronological age and as socioeconomic status decreases (social class and educational level). Additionally, lower values for both scales are observed in sedentary individuals, those with low adherence to the Mediterranean diet, or those who regularly consume alcohol.

Table 4 displays the prevalence of high values for vascular age (VA) and heart age (HA) according to various sociodemographic variables and healthy habits. A similar trend to that described for the mean values is observed: higher prevalence with increasing age, lower socioeconomic status, sedentary behavior, low adherence to the Mediterranean diet, and habitual alcohol consumption.

Table 5, which presents the results of the multinomial logistic regression, shows that all analyzed variables increase the risk of high VA and HA values. Age is the variable with the greatest influence. Low adherence to the Mediterranean diet doubles the risk of high HA and increases the risk of high VA slightly more.

Table 6 and Table 7 display the results of the longitudinal retrospective study for both sexes. It is observed that the difference in the prevalence of high HA and VA values between the pre- (2009) and post- (2019) periods increases with age and decreases with lower social class and educational level. This difference is also greater among smokers, habitual alcohol consumers, sedentary individuals, and those with low adherence to the Mediterranean diet.

## 4. Discussion

The mean values of HA and VA, as well as the prevalence of high HA and VA values, are higher among individuals of advanced age, lower socioeconomic status, smokers, habitual alcohol consumers, sedentary individuals, and those with low adherence to the Mediterranean diet.

Vascular and cardiac age represent biological metrics that provide insights into cardiovascular health beyond chronological age. Vascular age reflects the condition of arteries, particularly their stiffness and plaque burden [28], while cardiac age reflects the functional and structural state of the heart [29]. Numerous factors influence these metrics, including age, sex, socioeconomic status (SES), physical activity, adherence to the Mediterranean diet, and alcohol consumption.

Chronological age is a primary determinant of vascular and cardiac age [1,30]. Aging is associated with endothelial dysfunction [31], arterial stiffening [32], and increased intimal-medial thickness [33], which contribute to an elevated vascular age [34]. Similarly, cardiac aging involves left ventricular hypertrophy [35], reduced diastolic function [36], and increased myocardial fibrosis [37]. Studies demonstrate a direct correlation between chronological age and markers of arterial stiffness [38], such as pulse wave velocity (PWV) [39], and cardiac metrics, such as left ventricular mass index (LVMI) [40]. Additionally, advanced age and female sex are associated with increased vascular and ventricular stiffness, even in the absence of cardiovascular disease. This combined stiffening may contribute to the higher prevalence of heart failure with preserved ejection fraction in older individuals, particularly women [41]. With aging, arterial stiffness correlates with ventricular stiffness, increasing sensitivity to systolic pressure and affecting ventricular filling. In centenarians without cardiovascular disease, low ventricular–arterial coupling values have been observed, especially in women, suggesting structural remodeling of the left ventricle and greater myocardial stiffness. The literature indicates a progressive increase in arterial elastance with age, which is related to arterial stiffness. Moreover, an age-related increase in left ventricular end-systolic stiffness, secondary to structural changes in the left ventricular wall, leads to a concomitant increase in end-systolic elastance. The simultaneous increase in both arterial elastance and end-systolic elastance is necessary to maintain ventricular–arterial coupling (arterial elastance/end-systolic elastance) within normal values [42,43]. These changes elevate the risk of cardiovascular events, underscoring the importance of early interventions to mitigate age-related vascular and cardiac decline [44].

Sex significantly influences vascular and cardiac aging trajectories [45]. Premenopausal women typically exhibit a lower vascular age compared to men of the same chronological age, attributed to the protective effects of estrogen [46]. Estrogen enhances endothelial function [47] by increasing nitric oxide bioavailability [48] and reducing oxidative stress [49]. However, postmenopause, women experience accelerated vascular aging, characterized by increased arterial stiffness [50] and a higher prevalence of atherosclerosis [51]. Conversely, men tend to exhibit a steady progression of vascular aging throughout life, driven by factors such as testosterone’s influence on lipid metabolism [52] and inflammatory processes [53]. Cardiac aging also differs by sex, with men showing greater left ventricular mass [54] and women demonstrating higher susceptibility to diastolic dysfunction in older age [55]. These differences highlight the necessity of sex-specific strategies in cardiovascular risk assessment and management.

Socioeconomic status (SES) profoundly affects vascular and cardiac age through multiple pathways [56], including access to healthcare [57], lifestyle behaviors [58], and psychosocial stress [59]. Individuals with lower SES are more likely to engage in unhealthy behaviors [60], such as poor dietary habits [61], physical inactivity [62], and smoking [63], which accelerate vascular aging [64]. Furthermore, chronic stress associated with financial insecurity and social disadvantages contributes to sympathetic overactivation and endothelial dysfunction [64]. Studies indicate that individuals from lower SES backgrounds have higher PWV [65] and greater arterial stiffness [66], reflecting advanced vascular age. Similarly, SES disparities are linked to adverse cardiac remodeling and higher prevalence of heart failure with preserved ejection fraction (HFpEF) [67]. Interventions addressing SES-related inequalities are critical for reducing cardiovascular health disparities.

These interventions could be implemented as workplace health promotion programs, effectively reaching a large number of workers and encouraging healthy lifestyle habits in an accessible and familiar environment. However, these programs should not be standardized; rather, they should be tailored to the specific needs of each professional group, considering their level of health literacy and numerical skills. Personalizing these interventions would ensure better understanding and adherence to preventive strategies, maximizing their impact. Furthermore, effective preventive communication, based on clear and comprehensible messages, along with improvements in health literacy levels, are essential tools for reducing inequalities and promoting greater equity in the fight against diseases. This approach would allow for a more effective response to the burden of non-communicable diseases and enhance the overall well-being of the working population [68,69].

Physical activity exerts profound benefits on vascular and cardiac health, effectively reducing biological aging metrics [70]. Regular aerobic exercise improves endothelial function [71], reduces arterial stiffness [72], and enhances arterial compliance [73], thereby lowering vascular age [74]. Mechanistically, exercise promotes nitric oxide production [75], reduces oxidative stress [76], and decreases inflammation [77]. Meta-analyses have demonstrated that individuals engaging in high levels of physical activity exhibit significantly lower PWV and carotid intima-media thickness compared to sedentary individuals [78]. Cardiac benefits of exercise include improved left ventricular function [79], reduced myocardial fibrosis [80], and enhanced cardiac output. Notably, exercise’s anti-aging effects on the cardiovascular system are dose-dependent, with moderate to vigorous intensity providing the greatest benefits [81]. Public health strategies should emphasize promoting physical activity to mitigate vascular and cardiac aging.

The Mediterranean diet, characterized by high consumption of fruits, vegetables, whole grains, nuts, olive oil, and moderate wine intake, is well-documented for its cardioprotective properties [82]. Adherence to this dietary pattern is associated with reduced vascular and cardiac age [83] through mechanisms such as improved lipid profiles [84], reduced inflammation [85], and enhanced endothelial function [86]. Key components, such as polyphenols and omega-3 fatty acids, mitigate oxidative stress and promote vascular health [87]. Longitudinal studies have shown that individuals following a Mediterranean diet have lower rates of arterial stiffness and atherosclerosis progression [88]. Additionally, the diet’s anti-inflammatory properties positively influence cardiac remodeling, reducing left ventricular hypertrophy [89] and fibrosis [90]. DM has been shown to facilitate the maintenance of telomere length, which implies a reduction in the risk of age-related diseases. At the molecular level, the mechanisms by which DM improves age-related disorders can be attributed to the reduction of inflammation, oxidative stress, and mitochondrial dysfunction, together with the activation of telomerase action, which produces a significant impact on telomere length dynamics [91]. Encouraging adherence to the Mediterranean diet represents a viable strategy for reducing cardiovascular aging and associated risks.

Alcohol consumption has a complex relationship with vascular and cardiac age, with effects dependent on the quantity and pattern of intake [92]. Light to moderate alcohol consumption, particularly red wine, has been associated with reduced vascular age due to polyphenols, such as resveratrol, which enhance endothelial function [93] and reduce arterial stiffness [94]. However, excessive alcohol intake accelerates vascular aging [95] through mechanisms including increased oxidative stress [96], inflammation, and hypertension [97]. Chronic heavy drinking is linked to adverse cardiac remodeling, such as left ventricular hypertrophy [98] and reduced ejection fraction [99], contributing to an advanced cardiac age. The J-shaped relationship between alcohol and cardiovascular aging underscores the importance of moderation and individualized guidance in alcohol consumption.

The interplay among age, sex, SES, physical activity, diet, and alcohol consumption creates a multifaceted influence on vascular and cardiac aging [100]. For instance, individuals of lower SES often have reduced access to healthy foods and safe environments for physical activity, compounding the effects of socioeconomic disadvantage on cardiovascular aging. Similarly, sex-specific differences in hormonal status interact with lifestyle factors; postmenopausal women adhering to a Mediterranean diet and engaging in regular physical activity may partially mitigate the accelerated vascular aging associated with estrogen decline. Understanding these interactions is crucial for designing holistic and personalized interventions to combat cardiovascular aging.

Addressing factors influencing vascular and cardiac aging has significant clinical and public health implications. Early identification of individuals at risk, using metrics such as PWV and echocardiographic parameters, allows targeted interventions to prevent cardiovascular events. Public health initiatives should prioritize promoting physical activity, improving dietary patterns, and addressing SES disparities to reduce the burden of cardiovascular disease. Moreover, sex-specific guidelines in cardiovascular risk assessment and management can optimize outcomes.

Further research is needed to elucidate the molecular mechanisms underlying the impact of lifestyle and socioeconomic factors on vascular and cardiac aging. Longitudinal studies examining the interplay of these factors across diverse populations can inform precision medicine approaches. Additionally, leveraging emerging technologies, such as artificial intelligence and wearable devices, may enhance the monitoring and modification of cardiovascular aging metrics in real time.

## 5. Strengths and Limitations

This study exhibits several notable strengths, including its extensive sample size of nearly 140,000 workers, establishing it as one of the largest investigations conducted globally within this population. Furthermore, it represents one of the pioneering efforts to evaluate vascular and heart age across diverse sociodemographic factors and health-related behaviors. The comprehensive analysis of variables—including sociodemographic and lifestyle factors—coupled with a longitudinal design, enhances the capacity to identify causal relationships.

Another significant strength lies in the use of validated questionnaires to assess physical activity levels and adherence to the Mediterranean diet. These instruments provide a practical, cost-effective, and reliable method for both evaluation and follow-up.

Nonetheless, the study has certain limitations. It excludes specific population groups, such as unemployed individuals, retirees, and those under 18 or over 69 years of age, which may constrain the generalizability of the findings to the broader population. However, the large sample size mitigates this limitation to some extent.

Additionally, as the participants were exclusively drawn from Spain, the findings may not be directly generalizable to other populations. Consequently, these results should be interpreted with caution when considering their applicability to different demographic or geographic contexts.

Although our categorical classification of socioeconomic status is based on the classification of the group of social determinants of the Spanish Society of Epidemiology, it only delimits three classes. This constitutes another limitation, as it has not determined the working conditions, precariousness, or risk to which they are subjected according to their profession.

Another limitation arises from the reliance on self-administered questionnaires, which are inherently susceptible to biases such as recall bias and social desirability bias. Future research could improve the robustness of findings by incorporating objective validation methods.

Finally, the study did not account for certain potential confounding variables, such as the presence of comorbidities or the use of pharmacological treatments, due to the unavailability of this information for analysis.

## 6. Conclusions

Vascular and cardiac aging depend on factors such as chronological age, sex—with accelerated aging observed in postmenopausal women—and socioeconomic status. Physical activity and adherence to the Mediterranean diet contribute to reducing vascular and cardiac age, whereas excessive alcohol consumption accelerates it. These factors interact in a complex manner, influencing biological aging and highlighting the importance of healthy habits in maintaining better cardiovascular health over time.

Our results show that many of the studied variables influencing vascular and cardiac age are modifiable factors, including smoking, physical activity, diet, excessive alcohol intake, and socioeconomic status. This knowledge is highly valuable for healthcare policymakers, as it enables the implementation of measures targeting these factors to help delay vascular and cardiac aging and improve the population’s quality of life.

## Figures and Tables

**Table 1:** Variables and scores used to calculate cardiac age.

**Men**											
Total Cholesterol (mg/dL)	Points	HDL-c (mg/dL)	Points	Glucose (mg/dL)	Points	Smokers	Points	BMI (Kg/m^2^)	Points	SBP/DBP mm Hg	Points
<130 130–145 146–160 161–175 176–190 191–199 200–239 >240	−5 −4 −3 −2 −1 0 1 2	<35 35–39 40–45 46–50 51–55 56–60 61–65 >65	2 1 0 −1 −2 −3 −4 −5	<70 70–80 81–90 91–99 100–109 110–125 >125 *	−5 −3 −2 0 2 3 5	Yes Non	5 0	<20 20–22.5 22.6–24.9 25–29.9 30–34.9 ≥35	−2 −1 0 1 2 3	<120/<80 120–139/80–89 140–159/90–99 ≥160/≥100 *	−3 0 2 3
**Women**											
Total Cholesterol (mg/dL)	Points	HDL-c (mg/dL)	Points	Glucose (mg/dL)	Points	Smokers	Points	BMI (Kg/m^2^)	Points	SBP/DBP mm Hg	Points
<130 130–145 146–160 161–175 176–190 191–199 200–239 >240	−5 −4 −3 −2 −1 0 1 2	<40 40–49 50–55 56–60 61–65 66–70 71–75 >75	2 1 0 −1 −2 −3 −4 −5	<70 70–80 81–90 91–99 100–109 110–125 >125 *	−5 −3 −2 0 2 3 5	Yes Non	5 0	<20 20–22.5 22.6–24.9 25–29.9 30–34.9 ≥35	−2 −1 0 1 2 3	<120/<80 120–139/80–89 140–159/90–99 ≥160/≥100 *	−3 0 2 3

(*) Or in treatment.

**Table 2:** Characteristics of the sample.

	Men *n* = 83,282	Women *n* = 56,352	
	Mean (SD)	Mean (SD)	*p*-Value
Age (years)	41.4 (10.7)	40.1 (10.4)	<0.001
Height (cm)	173.8 (7.1)	161.2 (6.5)	<0.001
Weight (kg)	83.2 (14.6)	66.3 (13.9)	<0.001
BMI (kg/m²)	27.5 (9.2)	25.5 (8.9)	<0.001
Systolic blood pressure (mmHg)	126.2 (15.9)	115.6 (15.7)	<0.001
Diastolic blood pressure (mmHg)	76.6 (10.9)	71.1 (10.7)	<0.001
Total cholesterol (mg/dL)	199.6 (38.6)	194.6 (36.9)	<0.001
HDL-cholesterol (mg/dL)	50.0 (7.7)	54.7 (9.2)	<0.001
LDL-cholesterol (mg/dL)	122.6 (37.4)	121.5 (37.1)	<0.001
Triglycerides (mg/dL)	133.8 (95.6)	90.8 (49.7)	<0.001
Glucose (mg/dL)	93.0 (25.4)	86.8 (18.1)	<0.001
	%	%	*p*-value
<30 years	15.1	18.0	<0.001
30–39 years	29.6	31.0	
40–49 years	30.2	30.3	
50–59 years	20.9	17.7	
60–69 years	4.2	3.0	
Social class I	7.5	13.6	<0.001
Social class II	23.8	32.1	
Social class III	68.7	54.1	
Elementary school	66.4	48.1	<0.001
High school	26.9	40.0	
University	6.7	11.9	
Non-smokers	66.8	67.9	<0.001
Smokers	33.2	32.1	
Non-physical activity	62.4	51.4	<0.001
Yes physical activity	37.6	48.6	
Non-Mediterranean diet	65.8	52.8	<0.001
Yes Mediterranean diet	34.2	47.2	
Non-alcohol consumption	67.3	84.4	<0.001
Yes alcohol consumption	32.7	15.6	

HDL, high-density lipoprotein; LDL, low-density lipoprotein; SD, standard deviation.

**Table 3:** Mean values of different cardiovascular risk scales according sociodemographic variables and healthy habits by sex.

		ALLY HA		ALLY VA
Men	*n*	Mean (SD)	*n*	Mean (SD)
<30 years	12,558	1.3 (4.9)	0	no
30–39 years	24,648	4.2 (6.7)	24,648	2.2 (6.8)
40–49 years	25,178	7.9 (8.1)	25,178	6.9 (10.2)
50–59 years	17,370	11.7 (7.9)	17,370	12.6 (11.2)
60–69 years	3528	11.8 (7.4)	3528	14.3 (10.1)
Social class I	6236	4.9 (7.5)	5294	4.8 (9.4)
Social class II	19,856	6.3 (8.1)	17,914	6.2 (10.2)
Social class III	57,192	7.1 (8.1)	47,516	7.6 (10.4)
Elementary school	55,306	7.3 (8.5)	45,816	7.9 (11.4)
High school	22,408	6.7 (7.9)	20,050	6.8 (9.9)
University	5568	5.2 (7.6)	4858	5.0 (9.5)
Non-smokers	55,618	4.2 (7.3)	48,220	3.4 (8.4)
Smokers	27,664	11.8 (7.2)	22,504	14.8 (9.9)
Non-physical activity	51,984	8.9 (7.9)	47,646	9.2 (10.7)
Yes physical activity	31,298	3.2 (7.0)	23,078	2.6 (8.0)
Non-Mediterranean diet	54,792	8.7 (8.0)	50,012	8.8 (10.6)
Yes Mediterranean diet	28,490	3.0 (7.0)	20,712	2.7 (8.1)
Non-alcohol consumption	56,022	5.8 (7.8)	45,012	5.6 (9.3)
Yes alcohol consumption	27,260	8.8 (8.2)	25,712	9.6 (11.5)
Women		Mean (SD)		Mean (SD)
<30 years	10,110	−2.0 (5.0)	0	no
30–39 years	17,460	−1.8 (7.7)	17,460	−2.0 (6.8)
40–49 years	17,094	2.8 (10.2)	17,094	1.3 (11.8)
50–59 years	9984	8.4 (10.6)	9984	9.7 (14.4)
60–69 years	1704	8.6 (9.9)	1704	11.1 (13.1)
Social class I	7632	−2.0 (7.7)	5512	−2.1 (9.0)
Social class II	18,112	0.5 (9.3)	15,162	0.8 (11.1)
Social class III	30,608	3.3 (9.9)	25,568	4.0 (12.6)
Elementary school	27,086	3.4 (10.0)	22,908	4.2 (12.4)
High school	22,574	0.7 (9.4)	18,478	1.0 (11.5)
University	6692	−2.1 (7.7)	4856	−2.3 (9.0)
Non-smokers	38,252	−0.6 (9.2)	31,908	−0.1 (11.1)
Smokers	18,100	6.5 (8.9)	14,334	7.3 (12.1)
Non-physical activity	28,962	5.5 (9.8)	25,836	6.4 (12.8)
Yes physical activity	27,390	−2.3 (7.7)	20,406	−3.1 (8.0)
Non-Mediterranean diet	29,764	5.1 (9.9)	26,396	6.1 (12.8)
Yes Mediterranean diet	26,588	−2.2 (7.8)	19,846	−2.9 (8.2)
Non-alcohol consumption	47,536	0.1 (8.9)	37,846	0.0 (10.0)
Yes alcohol consumption	8816	10.0 (9.5)	8396	12.4 (14.2)

ALLY, avoidable lost life years; HA, heart age; VA, vascular age; SD, standard deviation.

**Table 4:** Prevalence of high values of different cardiovascular risk scales according sociodemographic variables and healthy habits by sex.

		ALLY HA High		ALLY VA High
Men	*n*	%	*n*	%
<30 years	12,558	1.8	0	no
30–39 years	24,648	10.1	24,648	3.2
40–49 years	25,178	28.2	25,178	14.9
50–59 years	17,370	47.8	17,370	33.3
60–69 years	3528	51.1	3528	41.0
Social class I	6236	15.4	5294	11.2
Social class II	19,856	22.2	17,914	14.9
Social class III	57,192	25.4	47,516	17.9
Elementary school	55,306	27.9	45,816	20.5
High school	22,408	23.1	20,050	15.5
University	5568	16.5	4858	11.8
Non-smokers	55,618	13.1	48,220	7.6
Smokers	27,664	45.7	22,504	36.1
Non-physical activity	51,984	32.3	47,646	21.9
Yes physical activity	31,298	10.0	23,078	5.7
Non-Mediterranean diet	54,792	31.4	50,012	21.0
Yes Mediterranean diet	28,490	9.5	20,712	6.0
Non-alcohol consumption	56,022	19.6	45,012	11.9
Yes alcohol consumption	27,260	32.9	25,712	25.0
Women	*n*	%	*n*	%
<30 years	10,110	0.8	0	no
30–39 years	17,460	5.0	17,460	2.0
40–49 years	17,094	18.5	17,094	10.2
50–59 years	9984	41.1	9984	30.1
60–69 years	1704	42.8	1704	39.4
Social class I	7632	5.5	5512	4.4
Social class II	18,112	12.4	15,162	9.2
Social class III	30,608	20.5	25,568	14.9
Elementary school	27,086	21.0	22,908	16.1
High school	22,574	12.8	18,478	10.1
University	6692	5.4	4856	4.4
Non-smokers	38,252	10.8	31,908	9.2
Smokers	18,100	26.6	14,334	19.6
Non-physical activity	28,962	26.3	25,836	20.0
Yes physical activity	27,390	4.8	20,406	2.8
Non-Mediterranean diet	29,764	25.6	26,396	19.5
Yes Mediterranean diet	26,588	5.0	19,846	3.1
Non-alcohol consumption	47,536	10.4	37,846	7.0
Yes alcohol consumption	8816	45.5	8396	37.1

ALLY, avoidable lost life years; HA, heart age; VA, vascular age.

**Table 5:** Multinomial logistic regression.

	ALLY HA High	ALLY VA High
	OR (95% CI)	OR (95% CI)
Women	1 *	1 *
Men	1.42 (1.37–1.47)	1.06 (1.04–1.08)
<30 years	1 *	no
30–39 years	1.33 (1.24–1.42)	1 *
40–49 years	4.28 (4.00–4.56)	1.81 (1.69–1.94)
50–59 years	17.67 (16.35–19.00)	7.00 (6.50–7.51)
60–69 years	114.91 (100.45–131.43)	34.48 (31.41–37.56)
Social class I	1 *	1 *
Social class II	1.74 (1.65–1.83)	1.77 (1.67–1.88)
Social class III	2.74 (2.23–3.25)	2.70 (2.38–3.03)
University	1 *	1 *
High school	1.35 (1.29–1.41)	1.48 (1.40–1.57)
Elementary school	1.41 (1.36–1.17)	1.98 (1.80–2.16)
Non-smokers	1 *	1 *
Smokers	2.88 (2.60–3.17)	2.95 (2.70–3.21)
Yes physical activity	1 *	1 *
Non-physical activity	2.33 (2.16–2.51)	3.38 (3.06–3.71)
Yes Mediterranean diet	1 *	1 *
Non-Mediterranean diet	2.02 (1.87–2.18)	2.40 (2.05–2.76)
Non-alcohol consumption	1 *	1 *
Yes alcohol consumption	2.06 (1.98–2.15)	3.20 (3.05–3.36)

ALLY, avoidable lost life years; HA, heart age; VA, vascular age; OR, odds ratio; CI, confidence interval. * Reference level.

**Table 6:** Longitudinal retrospective study in men.

		ALLY HA High		ALLY VA High	
Men	*n*	% PRE-% POST	Difference (%)	% PRE-% POST	Difference (%)
Social class I	1900	15.2–17.4	14.3	11.2–12.7	13.6
Social class II	5769	22.1–26.3	18.9	15.0–17.7	17.8
Social class III	16,560	24.9–30.0	20.3	17.6–21.2	20.6
Elementary school	16,022	24.6–29.7	20.6	17.3–20.9	20.8
High school	6501	22.5–26.7	18.6	15.4–18.1	17.6
University	1706	15.3–17.6	15.2	11.4–12.9	13.5
Non-smokers	16,244	12.8–14.3	11.4	7.4–8.2	10.6
Smokers	7985	45.1–58.0	28.6	36.1–47.0	30.2
Non-physical activity	15,045	31.8–36.5	14.8	21.7–28.2	29.8
Yes physical activity	9184	9.8–12.4	26.3	5.6–6.4	14.4
Non-Mediterranean diet	15,866	30.9–35.7	15.6	20.8–26.8	28.8
Yes Mediterranean diet	8363	9.3–11.6	24.8	5.8–6.7	15.5
Non-alcohol consumption	16,258	19.0–16.6	14.5	11.7–13.4	14.9
Yes alcohol consumption	7971	32.5–44.3	36.4	24.6–32.6	32.5

ALLY, avoidable lost life years; HA, heart age; VA, vascular age; PRE year 2010; POST year 2020.

**Table 7:** Longitudinal retrospective study in women.

		ALLY HA High		ALLY VA High	
Women	*n*	% PRE-% POST	Difference (%)	% PRE-% POST	Difference (%)
Social class I	2128	6.1–6.7	10.0	4.9–5.5	11.2
Social class II	5290	11.4–13.0	13.8	8.8–10.2	15.8
Social class III	8784	20.3–24.1	18.6	16.0–19.2	20.2
Elementary school	7836	20.6–24.5	19.0	15.7–19.0	21.0
High school	6518	12.1–13.8	14.3	10.1–11.6	14.9
University	1848	6.0–6.6	9.5	4.4–4.9	11.0
Non-smokers	10,992	10.8–11.7	8.6	9.2–10.0	8.9
Smokers	5210	25.5–30.7	20.4	19.2–24.4	27.2
Non-physical activity	8327	25.7–31.6	22.9	19.8–25.1	26.6
Yes physical activity	7875	4.7–5.1	7.7	3.0–3.3	10.3
Non-Mediterranean diet	8632	24.9–30.3	21.8	19.1–24.0	25.8
Yes Mediterranean diet	7570	4.8–5.2	8.6	3.2–3.6	11.7
Non-alcohol consumption	13,707	10.3–11.4	10.2	7.0–7.8	10.4
Yes alcohol consumption	2495	44.4–57.1	28.5	36.8–28.3	29.9

ALLY, avoidable lost life years; HA, heart age; VA, vascular age; PRE year 2010; POST year 2020.

## Data Availability

Data are not available due to ethical or privacy restrictions. This study’s data are stored in a database that complies with all security measures at the ADEMA-Escuela Universitaria. The Data Protection Delegate is Ángel Arturo López González.
